# Relationships between work outcomes, work attitudes and work environments of health support workers in Ontario long-term care and home and community care settings

**DOI:** 10.1186/s12960-018-0277-9

**Published:** 2018-03-22

**Authors:** Whitney Berta, Audrey Laporte, Tyrone Perreira, Liane Ginsburg, Adrian Rohit Dass, Raisa Deber, Andrea Baumann, Lisa Cranley, Ivy Bourgeault, Janet Lum, Brenda Gamble, Kathryn Pilkington, Vinita Haroun, Paula Neves

**Affiliations:** 1 Institute for Health Policy, Management and Evaluation, University of Toronto, 155 College Street, 4th Floor, Toronto, ON M5T 3M6 Canada; 2 Canadian Centre for Health Economics, University of Toronto, 155 College Street, 4th Floor, Toronto, ON M5T 3M6 Canada; 3 School of Health Policy & Management, York University, 4700 Keele Street, Toronto, ON M3J 1P3 Canada; 4 Faculty of Health Sciences, McMaster University, 1280 Main Street West, Hamilton, ON L8S 4K1 Canada; 5 Lawrence S. Bloomberg Faculty of Nursing, University of Toronto, 155 College Street, Suite 130, Toronto, ON M5T 1P8 Canada; 6 Telfer School of Management, 55 Laurier Ave E., Ottawa, ON K1N 6N5 Canada; 7 Department of Politics and Public Administration, Faculty of Arts, Ryerson University, 350 Victoria St., Toronto, ON M5B 2K3 Canada; 8 Faculty of Health Sciences, University of Ontario Institute of Technology, 2000 Simcoe Street, North Science Building, Oshawa, ON L1H 7K4 Canada; 9 Ontario Association of Non-Profit Homes & Services for Seniors, 7050 Weston Rd, Woodbridge, ON L4L 8G7 Canada; 10 Ontario Long Term Care Association, 425 University Avenue, Suite 500, Toronto, ON M5G 1T6 Canada; 11 Regional Geriatric Program of Toronto, 2075 Bayview Avenue, Toronto, ON M4N 3M5 Canada

**Keywords:** Work psychology, Health workforce psychology, Health support workers, Work environment, Perceived organizational support, Work attitudes, Job satisfaction, Work outcomes, Elder care, Home and community care, Long term care

## Abstract

**Background:**

Our overarching study objective is to further our understanding of the work psychology of Health Support Workers (HSWs) in long-term care and home and community care settings in Ontario, Canada. Specifically, we seek novel insights about the relationships among aspects of these workers’ work environments, their work attitudes, and work outcomes in the interests of informing the development of human resource programs to enhance elder care.

**Methods:**

We conducted a path analysis of data collected via a survey administered to a convenience sample of Ontario HSWs engaged in the delivery of elder care over July–August 2015.

**Results:**

HSWs’ work outcomes, including intent to stay, organizational citizenship behaviors, and performance, are directly and significantly related to their work attitudes, including job satisfaction, work engagement, and affective organizational commitment. These in turn are related to how HSWs perceive their work environments including their quality of work life (QWL), their perceptions of supervisor support, and their perceptions of workplace safety.

**Conclusions:**

HSWs’ work environments are within the power of managers to modify. Our analysis suggests that QWL, perceptions of supervisor support, and perceptions of workplace safety present particularly promising means by which to influence HSWs’ work attitudes and work outcomes. Furthermore, even modest changes to some aspects of the work environment stand to precipitate a cascade of positive effects on work outcomes through work attitudes.

## Background

Health support workers (HSWs; also called personal support workers or healthcare aides) deliver care to older Canadians in their homes and communities [1, 2] and in long-term care (LTC) homes (also called nursing homes) [3]. In Canada, they are an increasingly significant component of the healthcare labor force. Between 80,000 and 100,000 unregulated HSWs are employed in Ontario, the most populous province of Canada, with 57% working in the LTC sector and 34% in the health and community care (HCC) sector [4]. The other 9% work primarily in acute care. HSWs provide up to 80% of direct care to elderly residents and clients [5]. In Canada, HSWs are unregulated and efforts to organize and track these workers, in the interests of engaging them in the continued development of their roles and contributing to evidence-informed discussions on health human resources, vary markedly across provinces and territories. In Ontario, recent efforts to develop a registry of HSWs were discontinued and there remains no means by which to communicate broadly to these workers, to solicit their inputs into discussions regarding the sustainability of elder care, or to engage them in research.

The role of HSWs was originally designed to be supportive—to assist with daily living activities like bathing, dressing, meal preparation and, in the HCC sector, to undertake other light household tasks. The role was intended to sustain the general health and wellbeing of the people that HSWs work with and enable their independent living [5]. In Ontario, and Canada, this role has changed alongside changing demographics, changes in health policy relating to elder care such as aging-at-home initiatives (e.g. [6]), and a gradual re-composition of the workforce to provide increasingly complex care [7]. As in other countries [8, 9], in Canada there are increasing concerns around the influence of increased work demands relating to elder care on the incidence of absenteeism, burnout and turnover among direct care workers [10]—particularly given our health system’s dependence upon these workers for the majority (over 80%) of direct elder care. Together, these changes and concerns have prompted calls to revisit the appropriateness of supervision and worker preparation [5, 11], to develop human resource strategies [12], and for bold system reforms [13]. To us, these calls underscore the value in understanding the *work psychology* of HSWs such that we can appreciate, and anticipate, the impacts of changes to their work environments and can inform the development of evidence-based health human resource strategies that support these workers in their vital role as providers of direct elder care, and contribute to the sustainability of that part of our health care system devoted to elder care.

The overarching objective of our study is broad in that we wish to contribute to an understanding of the work psychology of HSWs where no prior comprehensive understanding exists. More specifically, the research question that we address here is: What are the relationships among perceptions of the work environment, work attitudes, and work outcomes of HSWs engaged in providing care to older Canadians in long-term care and home and community care settings in Ontario, Canada? This question has relevance to these workers, to their clients/residents, and to managers and policy decision makers engaged in LTC. To the best of our knowledge, a holistic examination of the concepts such as that on which we report here has not been undertaken in long-term care, or as it relates to HSWs.

### Overview of the literature on work psychology

Work psychology originates in the field of industrial-organizational (I-O) psychology which emerged in the late 1800s [14]. The majority of work focusing on work psychology has been conducted in settings other than health care; however, an increasing number of studies in “health workforce psychology” focus on the work psychology of healthcare workers [8, 15–18] and are motivated by an acknowledgement of the importance of the health and well-being of the health workforce not only to the quality of care and lives of those receiving care but to the general resilience and strength of health workers and health systems globally [9, 19]. The general work psychology research examines human behaviors in the workplace in the interests of creating work environments that motivate workers and support positive work outcomes [20, 21], acknowledging that the influence of work environments on worker attitudes and performance can be profound. We draw on these literatures—work psychology and health workforce psychology—to frame our study, with the objective of furthering our understanding of the work psychology of HSWs [22] and generating novel insights about the concept dynamics of work environments, work attitudes, and work outcomes (see [23]).

**Fig. 1:**
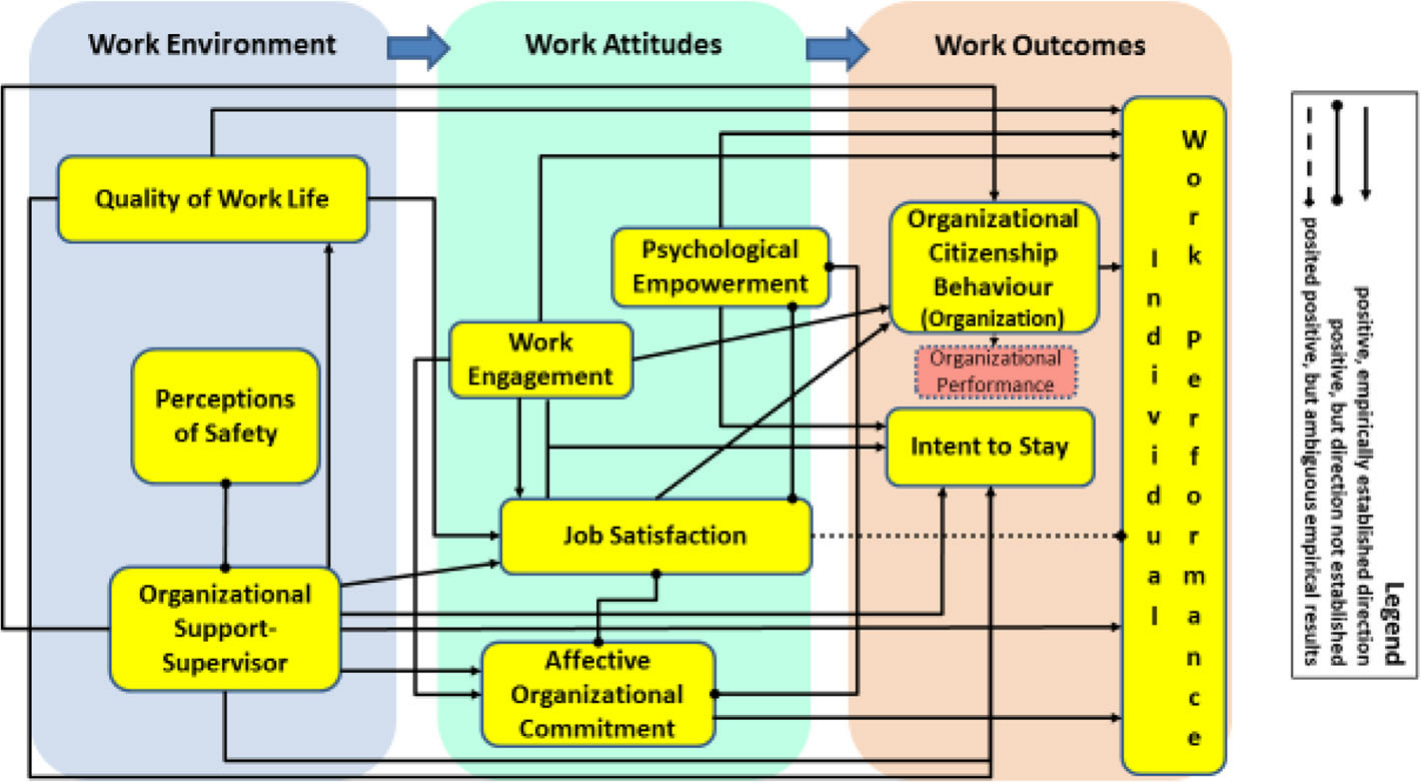
Original conceptual framework

The *Theory of Reasoned Action* [24] is the dominant theoretical foundation for work in work psychology. The main tenets of this theory are that work outcomes, like the act of remaining in a job (or the converse act of leaving a job), or in-role and extra-role behaviors, are directly determined by behavioral intentions. Those intentions, and subsequent behaviors, are influenced by perceptions of the work environment [16] that workers develop through interactions with their leaders/supervisors and co-workers [21] and the quality of their work lives [25]. The influence of the work environment on intentions and behaviors is understood to be chiefly indirect through intervening work attitudes. “Proximal” attitudes to behavioral intentions include job satisfaction and organizational commitment [8, 17, 26]. More “distal” work attitudes include work engagement [27] and psychological empowerment [28]. Figure 1 presents an original conceptual framework that we developed to guide our study, based on our review of the work psychology and health workforce psychology literatures.

#### Work environment

*Perceived organizational support* (POS) is widely studied [18, 21, 29, 30]. These perceptions are formed based on an organization’s actions, signaling to workers that their contributions are valued and that their employers are concerned for their wellbeing. Supervisors are agents of an organization and their actions are important to workers’ perceptions of *supervisor support.* Such perceptions can lead to engagement in reciprocal worker-supervisor “exchange” relationships [21]. POS has strong positive associations with affective organizational commitment and job satisfaction (work attitudes) [18] and with in-role and extra-role behaviors that positively influence individual worker performance outcomes [30]. POS is associated with decreases in negative work behaviors such as withdrawal and turnover [21]. POS is also associated with other perceptions of the work environment, *quality of work life* (QWL) [21] and *perceptions of workplace safety* [31]. Organizational actions to ensure worker safety may be interpreted by workers as evidence of the organization’s concern for their safety and wellbeing.

QWL perceptions arise from the satisfaction of workers’ needs through participation at work [25, 32]. If survival needs, social needs, ego needs, and self-actualization needs are met through workers’ jobs, effects can spill over to other aspects of workers’ lives, including satisfaction with family and social relationships and positive views of physical and mental health and wellbeing [25]. In addition to positive associations with POS, QWL is positively associated with the work attitudes of organizational commitment and job satisfaction and with the work outcomes of individual productivity/performance and intent to stay [15, 25].

#### Work attitudes

Attempts over the past few decades in I-O psychology to explain variation in work outcomes at the individual level have been frustrated. This is now generally attributed to a failure of past studies to consider intermediary work attitudes that are sensitive to the vagaries of working conditions and to interactions with leaders and co-workers [33, 34]. Four work attitudes are suggested as important: work engagement, affective organizational commitment, job satisfaction, and psychological empowerment.

*Work engagement* is “a positive, fulfilling work-related state of mind that is characterized by vigor, dedication, and absorption” ([27], p. 702). Vigor relates to high energy levels applied to work and to mental resilience. Dedication refers to work enthusiasm and inspiration. Absorption refers to being engrossed in one’s work. Work engagement relates positively and directly to job satisfaction and to in-role [35] and extra-role behaviors like organizational citizenship behaviors [20]. It is associated negatively with withdrawal behaviors including turnover intention [27].

Work engagement is associated with another work attitude, *affective organizational commitment*, which is the positive affect and affiliation that workers develop for their organizations [26]. Both work engagement and organizational commitment are significantly and positively associated with work performance. Workers with high commitment and engagement pursue the goals and interests of the organization over their own and diligently perform their assigned work roles [26].

Extensive work in I-O psychology has sought to determine the nature of the relationship between *job satisfaction* and individual performance, but net results are ambiguous (dotted line in Fig. 1) [36]. Job satisfaction is associated positively with both organizational commitment [26] and with psychological empowerment [37, 38].

*Psychological empowerment* refers to feeling capable of shaping one’s work role and context [28] and is composed of four factors: meaning associable to work tasks, feelings of competence and belief in one’s abilities to perform work activities, self-determination relating to control and choice over work behaviors, and observable positive impacts of one’s work behaviors. It relates positively to other attitudes including job satisfaction [30] and organizational commitment [39], and is linked to work outcomes including (lower) turnover intention and (elevated) worker performance such as effectiveness [40] and productivity [28, 40].

#### Work outcomes

*Organizational citizenship behaviors* (OCBs) are extra-role behaviors that include organizational loyalty and helping [41]. One OCB dimension, individual initiative, is distinct from all others. It refers to “voluntary acts of creativity and innovation” comprising “constructive efforts by individuals to identify and implement changes with respect to work methods, policies, and procedures” to improve organizational effectiveness ([42], p. 469). This dimension is referred to as OCB-O (“O” for “organization,” the intended beneficiary). It is particularly interesting to management/I-O scientists because OCB-Os are linked empirically to workplace proactivity, creativity, innovation, task performance and job satisfaction [43] and have been shown to influence organizational performance outcomes positively in many different industries [42]. Because they are aimed at improving organizational effectiveness, OCB-Os may be especially important in resource-constrained environments such as LTC and HCC settings.

We consider *intent to stay* because its corollary, turnover intention, is an acknowledged issue among HSWs in some jurisdictions [1, 44]. Intent to stay is known to be influenced by the work environment, particularly by perceived supervisor support [45]. In diverse work settings, including healthcare, turnover intention has been related directly and negatively to job satisfaction [17] and to withdrawal behaviors that are negatively linked to *individual work performance*, including effectiveness and productivity [28].

## Methods

Over July–August 2015, we collected data on the measures identified in Fig. 1 (excluding organizational performance) via a survey administered to a convenience sample of HSWs working in Ontario LTC homes and HCC agencies. A cross-sectional survey design was used as it is appropriate for our exploratory study.

The research project of which this manuscript is part has been approved by the University of Toronto’s Human Research Ethics Program Protocol Reference 30742.

### Survey development

While measures and scales with good psychometric properties exist for most of the concepts in our framework (see “Measures” section below), a few had not been used in healthcare settings or had not been used in LTC and HCC settings. We therefore undertook extensive development work in the form of psychometric testing to prepare the measures for three constructs for inclusion in the survey: work engagement, psychological empowerment, and OCB-O [46].

### Measures

Table 1 briefly describes the measures included in our survey. Measures for work engagement, psychological empowerment, OCB-Os, and job satisfaction were identical to those used in our pilot survey [46] and we used well-established measures for QWL, organizational support-supervisor, perceptions of workplace safety, organizational commitment, and intent to stay. We developed a context-specific self-reported measure for quality of care [47].

**Table 1:** Measures of work environment, attitudes and outcomes - *HSW Worklife Survey*

	Concept	Measure source
Work environment	Quality of work life	Quality of Work Life Measure (13 items) [25]
	Organizational support-supervisor	Two items based on those for Supervisor Support from 8-item Survey of Perceived Organizational Support (SPOS) [29]
	Perceptions of workplace safety	Seven items from the 29-item Western Health Risk Assessment Screening Tool (WHRAST) [61]
Work attitudes	Work engagement	Utrecht Work Engagement Survey-9 (UWES-9) (9 items) [27]
	Organizational commitment	Organizational Commitment Questionnaire (OCQ) (9 items) [62]
	Job satisfaction	Sub-scale from the Michigan Organizational Assessment Questionnaire (MOAQ-JSS) (3 items) [63]
	Psychological empowerment	Psychological Empowerment Instrument (12 items) [40]
Work outcomes	Intent to stay	Global measure (modeled on [23], 1 item)
	Organizational citizenship behaviors—organization	Measures of OCB-O (4 items) [42]
	Individual work performance	Context-specific; developed in consultation with collaborators (2 items: “I feel confident in my ability to provide an *acceptable level* of care to my clients/residents; I feel confident in my ability to provide *high quality* care to my residents/clients”.)

The survey also collected data on a broad set of additional questions including demographic characteristics and work history.

### Sample and survey distribution

Ours was necessarily a convenience sample, since there is no direct means by which to engage this worker population in research (e.g., through a worker registry). The survey was administered through our industry collaborators who represent HCC agencies (Home Care Ontario) and LTC homes (the Ontario Association of Non-Profit Homes & Services for Seniors, and the Ontario Long Term Care Association) operating in Ontario. Interested agencies and LTC homes responded to an invitational email from collaborators and requested paper or electronic surveys. Our collaborators forwarded our message, addressed to HSWs engaged in elder care with an embedded link to the e-survey, directly to interested agencies and operators who then forwarded the message to their HSWs. Requests for paper surveys were met by mailing paper copies directly to LTC homes and HCC agencies. We followed a modified Dillman approach [48, 49] appropriate for self-administered surveys, with reminder e-messages sent to participants (via our collaborators through agencies and operators) at 1, 3 and 4 weeks after the first invitational e-message. All survey communications were developed with our collaborators to enhance salience, completion, and response rates [50]. A random draw for several prizes such as gift cards was available to participants who completed the survey; contact information for the draw was collected separately from the completed surveys.

### Data collection and coding

Respondents to the paper survey returned it anonymously by mail, and responses were double-entered as they were received. Responses to the electronic survey were captured automatically and anonymously through *FluidSurveys*. There were no missing data among the electronic responses, and missing data ranged from 2.2 to 22.2% for responses to items on the paper survey. Given the steps that we took to minimize missing data, we treated missing data as missing. For paper surveys, we used pairwise deletion to preserve as many responses as possible.

### Data analysis

LISREL 9.10 was used to generate descriptive statistics (PRELIS), including Spearman correlations, and to complete factor analyses. We used exploratory path analysis to examine the inter-relationships among the concepts. This is an appropriate analytic approach because these concepts are under-explored in these work contexts and unexplored in a single model. Path analysis obviates the need for mediation analysis using separate regressions [51]. We modeled work environment variables as predictor variables, work attitude variables as mediators, and work outcome variables as outcome variables.

PRELIS tests of univariate normality supported the use of maximum likelihood estimation [51]. For the overall model, we use the fit statistics suggested by Hu and Bentler [52]. Acceptable model fit is indicated by a non-significant *χ*^2^ value, a comparative fit index > 0.90, a Tucker-Lewis index > 0.90, an incremental fit index > 0.90, and root mean square standard error of approximation < 0.08. All our fit indices meet “acceptable” cut-off values and meet “good” cut-offs [52]. Because our study is exploratory, we examined the modification indices generated for additional relationships that were not suggested by our review of extant literature.

## Results

### Response rate

A total of 1616 surveys were requested or accessed: 180 paper surveys sent to HCC agencies; survey link accessed by 170 HSWs employed by HCC agencies; 600 paper surveys sent to LTC homes; and survey link accessed by 666 HSWs employed by LTC homes. Of the total, 460 of the surveys were usable: 183 electronic surveys (88 from HCC and 95 from LTC) and 277 paper surveys (96 from HCC and 181 from LTC). This gave a 28.5% response rate. This is a modest response rate, but the number of respondents is more than sufficient for our path analysis [53]. Nor was it unexpected given the indirect means of survey communication and administration, and the potentially sensitive nature of the survey questions (such as feelings about one’s workplace, adequacy of supervisor support).

### Respondents

Table 2 summarizes respondent characteristics, which are comparable to those observed by other researchers who have examined this worker population in Ontario [5], and to our pilot survey [46].

**Table 2:** Respondent characteristics—*HSW Worklife Survey*

Respondent characteristics	% total sample *N* = 460
Age	
< 24–29 years	13.2%
30–39 years	16.7%
40–49 years	26.2%
50–59 years	35.4%
> 60 years	8.6%
Female	92.6%
Immigrant to Canada	35.3%
English as second language	45.5%
Work in LTC/HCC	59.4%/40.6%
Work experience	
< 1 year	4.6%
1–10 years	46.6%
11–20 years	33.0%
> 20 years	15.9%
Training and education	
Grade school	1.8%
High hchool	24.9%
College	62.8%
University	10.4%
Years with employer	
< 1 year	4.2%
1–9 years	48.6%
10–19 years	25.7%
20–35 years	12.7%
Unanswered	4.2%
Type of work	
Full-time	43.8%
Part-time	17.9%
Casual	3.6%

### Sample representativeness

As discussed above, there is no direct means by which to engage the HSW population in research in Ontario. No registry of these workers exists in Ontario, and because we were reliant on our industry collaborators for survey distribution, we cannot say that the population was sampled randomly. Further, because of uncertainties around estimates of the size of the workforce population in Ontario [5, 12], which persists given the lack of a registry and is exacerbated in part by the fact that these workers are unregulated, we are further constrained in our ability to comment on the representativeness of our sample. We note that the gross-grained characteristics of our survey respondents are similar to those noted by others [4, 5].

### Scale reliabilities and factor analysis

We undertook reliability testing and factor analyses to compare our findings with other studies. Our confirmatory factor analyses for OCB-O (one factor), affective organizational commitment (one factor), work engagement (one factor), and psychological empowerment (four factors) support measurement models that are consistent with those established in the literature and in our earlier pilot study [46]. Instead of a seven-factor model [25], we find support for a three-factor model of the QWL with factors relating to needs for self-actualization, work-life balance, and feelings of value and self-esteem. Exploratory factor analysis supports a one-dimensional model for perceptions of workplace safety. We did not complete factor analyses for organizational support-supervisor, intent to stay, job satisfaction, or self-reported individual work performance since these measures had only 1 to 3 items. All measurement models for the factor analyses meet the fit criteria for large sample sizes with dependence among common and error variances, and all are “acceptable” with most indices at “good” levels [52]: standardized root mean squared residual < 0.08; comparative fit index > 0.90; and Bentler-Bonnett Index or normed fit index > 0.90. All scales had acceptable reliability with Cronbach’s alpha > 0.7.

### Descriptive statistics

With the sole exceptions of the correlations between OCB-Os and perceptions of workplace safety, OCB-Os and intent to stay, and psychological empowerment and intent to stay, all correlations are significant and positive (Table 3). The correlation coefficients are all of reasonable magnitude.

**Table 3:** Spearman correlations, scale means and standard deviations—*HSW Worklife Survey*

Measure (scale)	*n*	Mean, SD	1	2	3	4	5	6	7	8	9	10
1. Organizational support-supervisor (1–5)	450	2.05, .95	1.0									
2. Perceptions of workplace safety (1–5)	425	2.82, .72	0.45**	1.0								
3. Organizational commitment (1–5)	425	2.09, .74	0.56**	0.48**	1.0							
4. Job satisfaction (1–5)	450	1.89, .80	0.42**	0.43**	0.69**	1.0						
5. Organizational citizenship behaviors-organization (1–7)	406	3.44, 1.03	0.20*	0.05	0.25**	0.20**	1.0					
6. Quality of work life (1–5)	358	2.62, .65	0.55**	0.55**	0.63**	0.62**	0.24**	1.0				
7. Intent to stay (1–4)	453	1.28, .72	0.16**	0.23**	0.35**	0.31**	0.08	0.31***	1.0			
8. Work engagement (1–7)	445	0.92, .95	0.40**	0.35**	0.48**	0.57**	0.20*	0.56**	0.24**	1.0		
9. Psychological empowerment (1–7)	366	2.69,1.08	0.23**	0.28**	0.35**	0.37**	0.45**	0.33**	0.09	0.25**	1.0	
10. Individual work performance (1–5)	449	1.58, .57	0.29**	0.24**	0.49*	0.46**	0.14**	0.33**	0.14**	0.26**	0.35**	1.0

N = 460, **p < 0.01, ***p < 0.001

Low mean scores indicate favorable responses. We retained the scales and question formats developed by the original instrument authors; therefore, three scales range from 1 (strongly agree) to 7 (strongly disagree), one from 1 to 4, and most from 1 to 5. Responses for perceived organizational support-supervisor, organizational commitment, and job satisfaction are generally positive with mean scores indicating “agree” to questions on perceptions of strong support, feelings of affective commitment, and experienced work satisfaction. For work engagement, the mean response is “always” to questions on being engaged and absorbed in work. The mean response for psychological empowerment is generally positive where HSWs “agree” that their work is meaningful and important and that they are good at their jobs. Mean responses for other work attitudes are less positive: responses for perceptions of workplace safety and QWL tend toward neutral (“neither agree nor disagree”). In terms of work outcomes, the mean score for OCB-Os tends toward neutral; the mean response for self-reported individual work performance is generally positive (respondents “agree” that they provide acceptable and high quality of care to residents/clients); and the mean response to the intent to stay question suggests that many workers do not intend to stay with their current employer beyond the next 6 months.

### Path analysis

Our path analytic model affords a good fit to the data [54], with *χ*^2^(22) = 48.564, *p* = 0.0009, comparative fit index = 0.990, Tucker-Lewis index/non normed fit index = 0.979, incremental fit index = 0.990, and root mean square standard error of approximation = 0.0512. Our model accounts for 17.6% of the variance in individual work performance, 21.2% of the variance in OCB-Os, and 12.7% of the variance in intent to stay.

**Fig. 2:**
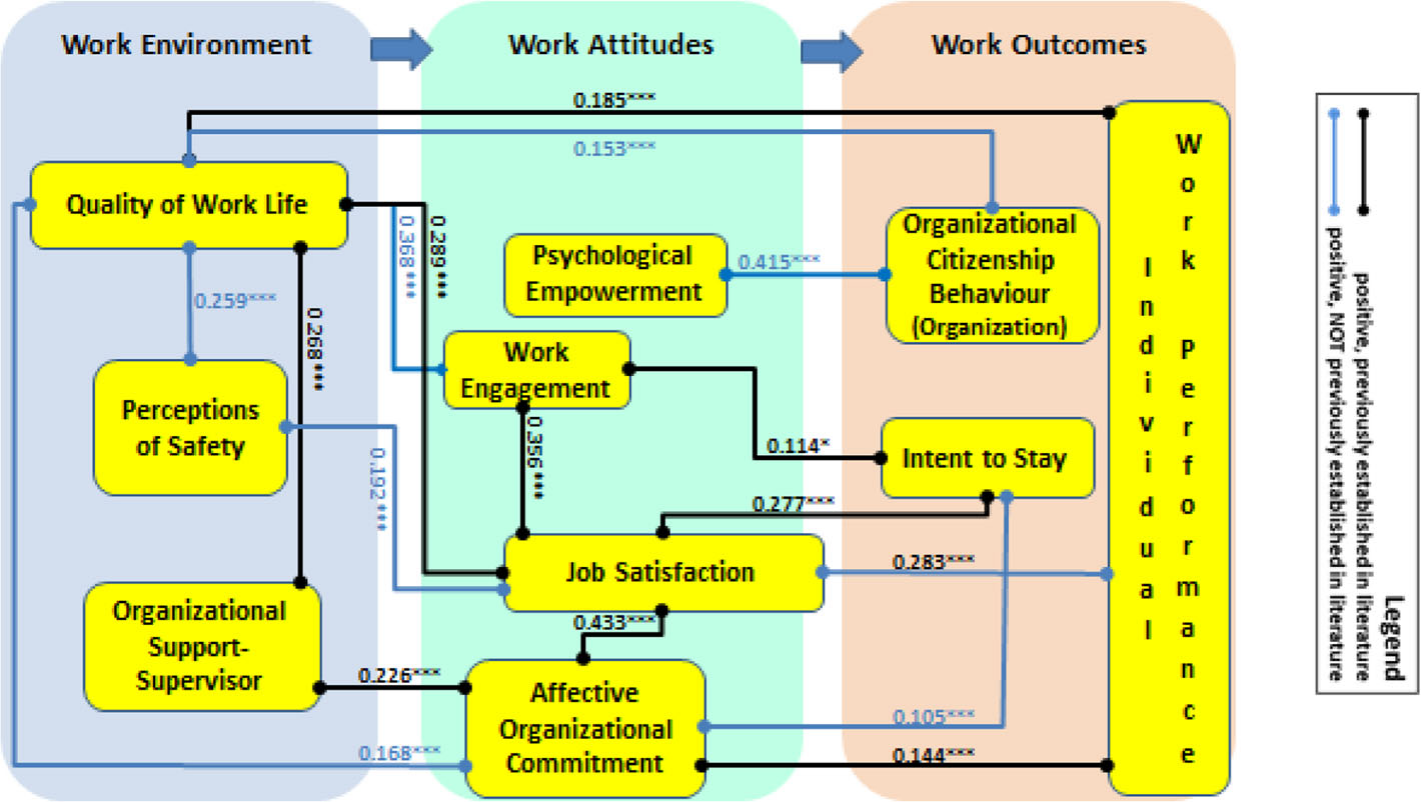
Re-conceptualized conceptual framework

Figure 2 is a re-conceptualization of our original conceptual framework (Fig. 1) that includes the significant standardized coefficients from our path analysis. Standardized coefficients permit comparisons of relative importance within the specific sample [55]. All of the associations that we observe are positive.

#### Work outcomes

OCB-Os are associated with QWL (0.153, *p* < 0.001) and strongly associated with psychological empowerment (0.415, *p* < 0.001). Intent to stay is significantly associated with three work attitudes: most strongly with job satisfaction (0.277, *p* < 0.001), followed by work engagement (0.114, *p* < 0.05), and affective organizational commitment (0.105, *p* < 0.001). Individual-level work performance is positively associated with two work attitudes, job satisfaction most strongly (0.283, *p* < 0.001) and organizational commitment (0.144, *p* < 0.001), and with one aspect of work environment, QWL (0.185, *p* < 0.001).

#### Work attitudes

Work attitudes are inter-related. Affective organizational commitment is more strongly associated with job satisfaction (0.433, *p* < 0.001) than with two measures of work environment, QWL (0.168, *p* < 0.001) and perceived organizational support-supervisor (0.226, *p* < 0.001) and with self-reported individual work performance (0.144, *p* < 0.001). Job satisfaction is most strongly associated with one other work attitude, work engagement (0.356, *p* < 0.001), and associated also with two measures of work environment, QWL (0.289, *p* < 0.001) and perceptions of workplace safety (0.192, *p* < 0.001).

#### Work environment

QWL is associated with the other two measures of work environment, perceived organizational support-supervisor (0.268, *p <* 0.001) and perceptions of workplace safety (0.259, *p <* 0.001). Job satisfaction (as above) and work engagement (0.368, *p <* 0.001) are associated with QWL with a relatively stronger association between work engagement and QWL.

## Discussion

In general, we find that work outcomes for HSWs are related to their work attitudes, which are in turn related to how they perceive work environments. The more nuanced relationships that we observe among specific aspects of HSWs’ work environments, work attitudes, and work outcomes reveal potential levers to influence work outcomes/performance in practical and positive ways. These might include initiatives that target work attitudes directly, and initiatives that influence them indirectly through (positive) changes that are effected by managers and human resource personnel to these workers’ work environments.

### Direct effects of work environment on work outcomes

Consistent with previous work [25], we observe significant positive relationships between QWL and individual work performance, and QWL and OCB-Os. Initiatives that focus on enhancing QWL for HSW may enhance work performance. Practically, these initiatives might include engaging staff in discussions of their aspirations and interests (actualization needs), and putting in place policies that assist them in balancing economic and family needs with their own health and safety. Our findings also suggest that QWL can be enhanced through improved perceptions of supervisor support and perceptions of workplace safety and that efforts to alter these other facets of HSWs’ work environments are likely to further amplify effects on individual work performance through the intermediary work attitudes of work engagement, job satisfaction and affective organizational commitment (discussed below).

### Mediating role of work attitudes

Three of the four work attitudes that we examine are important mediators between HSWs’ work environment and work outcomes. Three of the four strongest associations that we observe in our analysis are the mediating relationships between the work attitudes of work engagement, job satisfaction, and affective organizational commitment and QWL and intent to stay. Others have found associations between affective organizational commitment and intent to leave [17] in Swiss nursing homes. All three of these mediating variables are related, with job satisfaction associated with both HSWs’ work engagement and organizational commitment. The fourth strong association is observed between psychological empowerment and OCB-Os. While we do not suggest developing initiatives that target work attitudes, because they are generally difficult to modify directly, what our findings do suggest is that initiatives on the part of managers that target the more modifiable aspects of work environment stand to exert real and positive effects—*through* work attitudes—on HSWs’ intent to stay, their individual work performance, and OCB-Os.

For example, supervisor support is known to influence overall POS [56]. In particular, support from inspirational leaders who provide workers with “purpose and efficacy” enhances workers’ affective commitment to their organization [21]. In some sense, this is reciprocation (“you like me so I like you”) and this feeling of positive affect manifests as workers positively directing their behaviors to organizational goals (“let me repay you for your concern and support with actions that will help you”). Supervisors of HSWs, then, may be a key means to influence their work performance. Leadership training and development that sensitizes supervisors to the dynamics between their behaviors and those of HSWs, and enhances their leadership skills, is one promising means by which HSWs’ performance may be influenced.

Perceptions of workplace safety are arguably the most readily modifiable aspect of the work environment that we consider. Workplace initiatives can be put in place that acknowledge the safety and health risks faced by HSWs in their work—ascertained through staff discussion or anonymous survey. Investments can be made in infrastructure to mitigate risks, along with training to equip HSWs and their supervisors with the skills to anticipate and manage risks. These initiatives stand to directly and positively enhance job satisfaction [44], which is positively and significantly associated with both intent to stay and individual work performance. Initiatives that improve HSWs’ perceptions of workplace safety may also enhance their perceived QWL, specifically the need to feel valued, assumedly through addressing their health and safety needs.

The association between job satisfaction and individual work performance, as discussed earlier, has long been a focus for I-O psychologists and management scientists and is the focus of studies in health workforce psychology (see [8]). We find a significant positive association. A good stock of knowledge exists on how to enhance job satisfaction through POS [21] and QWL [25] initiatives. POS for HSWs may be enhanced by human resource policies and practices that are unbiased, equitable and consistently and openly applied, such as formal policies around shift work assignments, work leaves, performance feedback and remuneration. In turn, these can increase job satisfaction and individual work performance.

### Influencing organizational citizenship behaviors

Only two significant pathways relate to OCB-Os. There is an association with the work environment concept QWL (discussed above) and an association with psychological empowerment which is among the four strongest associations observed in our analysis. In both instances, OCB-Os may be expressed in exchange for employing organizations meeting workers’ needs. While psychological empowerment is a work attitude, it may represent a need on the part of HSWs to experience meaning in their work, to feel competent, to act independently, and to feel that they are “making a difference.” In short, expressions of OCB-Os are related importantly to HSWs’ sense of efficacy and value. These perceptions can be formed or enhanced by actions that communicate HSWs’ value to their organization and that reinforce HSWs’ feelings of worth and self-esteem. OCB-Os among HSWs might be enhanced by human resource policies and programs that focus on public recognition for jobs well done; work-life balance; initiatives on the part of managers that empower HSWs to act independently to solve workplace problems [57]; and supervisor/leadership training to develop mentorship and feedback skills.

#### Study limitations

There is no means of directly engaging the HSW population in Ontario: HSWs are unregulated and no registry of these workers exists. Of necessity therefore, we used an indirect means to access HSW respondents: our response rate reflects this and we cannot be confident that our sample is representative or random. That said, our respondents have characteristics that are similar to those observed in other studies of these workers [4, 5, 12]. Our use of self-report survey data exposes us to common methods bias [58]. Although gauging and addressing worker perceptions are what is important in understanding work psychology, future research might usefully include additional data sources for individual work performance.

#### Theoretical contributions

To our knowledge, no previous empirical studies have simultaneously considered all the concepts that we examine here; hence, our findings advance general understanding of the complex concept dynamics of work psychology.

## Conclusions

While their importance to the sustainability of elder care is increasingly acknowledged [9], these workers are historically understudied in Canada—in part because they are unregulated and, in most provinces and territories, unrepresented in the health workforce. An expanding scope of work and increasing demands for more complex care are suggested as factors in the incidence of burnout, absenteeism and turnover among these workers in Canada and other countries [8–10]. Exploring these relationships is the purview of work psychology, and health workforce psychology, researchers. Our study offers several novel and timely insights into the nature of HSWs’ work psychology. Arguably, all aspects of the work environment that we examined (perceptions of organizational support-supervisor, perceptions of workplace safety, and QWL) are within the power of management to modify; however, our analysis suggests that those that we highlight above—QWL, perceptions of supervisor support, and perceptions of workplace safety—offer particularly promising means by which to influence HSWs’ work attitudes and work outcomes. Even modest modifications to some aspects of the work environment could precipitate a cascade of positive effects. Both LTC and HCC are sectors in which “visibility” is low for HSWs, their residents/clients, and episodes of care. Our findings raise this visibility. We have insights now into how HSWs’ perceptions of their work environments influence how they feel about their work, and how these work attitudes in turn influence their work behaviors and performance. HSWs play a critical role in elder care; these early insights into their work psychology lay a foundation for further studies, ultimately leading us to respond in an informed and effective way to their work-related needs. This may go some way toward addressing system sustainability concerns about the care of older adults [59, 60].

## Abbreviations

HCC: Health and community care
HSW: Health support workers
I-O: Industrial-organizational
LTC: Long-term care
OCB: Organizational citizenship behaviors
OCB-O: Organizational citizenship behaviors - organization
POS: Perceived organizational support
QWL: Quality of work life
